# Ultrasound-guided serratus posterior superior muscle block for myofascial pain syndrome in the cervicoscapular region: a report of three cases

**DOI:** 10.1186/s40981-025-00807-7

**Published:** 2025-07-29

**Authors:** Atsushi Sawada, Michiaki Yamakage

**Affiliations:** Department of Anesthesiology, Sapporo Medical University School of Medicine, South 1 West 16 Chuo-ku, 060-8543 Sapporo, Japan

**Keywords:** Serratus posterior superior muscle block, Myofascial pain syndrome, Cervicoscapular pain, Interfascial block

## Abstract

**Background:**

These case reports focus on successful pain management with ultrasound-guided serratus posterior superior muscle (SPSM) block using 30 mL of 0.25% ropivacaine or physiological saline in three myofascial pain syndrome (MPS) patients presented with cervicoscapular pain.

**Case presentation:**

The SPSM block was administered to three ambulatory patients (cases #1, #2, and #3) who presented with cervicoscapular pain. The SPSM block with 30 mL of 0.25% ropivacaine drastically decreased an NRS score and provided 2–3 weeks of pain relief in cases #1 and #2. On the contrary, the SPSM block with 30 mL of physiological saline also mildly decreased an NRS score and provided 3 weeks of pain relief in cases #1 and #3.

**Conclusions:**

The SPSM block using 30 mL of 0.25% ropivacaine or physiological saline successfully decreased the NRS scores in three MPS patients. These findings suggest that the SPSM block may serve as a useful therapeutic option in MPS patients presenting with cervicoscapular pain.

## Background

Clinical guidelines for the management of myofascial pain syndrome (MPS) recommend pharmacological treatments, physical therapy, and interventional procedures including multiple trigger point injections [1]. However, some patients do not respond adequately to these treatments. Interfascial blocks, with injection of local anesthetics or physiological saline between the interfascial spaces, have been reported to reduce the intensity of pain in MPS patients [2, 3]. Taketa et al. reported that the serratus posterior superior muscle (SPSM) block using 15 mL of 0.25% ropivacaine alleviated cervicoscapular pain in 15 patients with MPS [4]. Recently, we have reported that the SPSM block probably provides pain relief by acting as interfascial block rather than a segmental nerve block, and the SPSM block using 30 mL of injectate volume tends to spread more widely compared to the SPSM block using 20 mL of the injectate in the cadaveric study [5]. Here, we present case reports of successful pain management with ultrasound-guided SPSM block using 30 mL of 0.25% ropivacaine or physiological saline in three MPS patients presented with cervicoscapular pain.

## Case presentation

The SPSM block was administered to three ambulatory patients who presented with cervicoscapular pain and were diagnosed with MPS according to the MPS diagnosis criteria [6]. The symptoms meeting diagnostic criteria for MPS in three patients are summarized in Table 1. Since all three patients did not complain of pain in the shoulder joint, we excluded the pathophysiology in the shoulder joint from the differential diagnosis. The patient characteristics, the diagnosis, and the details of pharmacological treatments are summarized in Table 2. Written consent was obtained from all three patients for publication of these case reports after informing them of the risks, including pneumothorax and systemic local anesthetic toxicity, and benefits of the procedures, as well as possible alternative treatments. Ultrasound-guided SPSM block was performed by the same experienced anesthesiologist (AS). The Numerical Rating Scale (NRS) of pain in the cervicoscapular region was assessed prior to the SPSM block administration and 60 min after the SPSM block administration. Sensory loss in the dermatomes between T1 ang T8 at the back was assessed using the cold test 60 min after the SPSM block administration. The pain relief period provided by the SPSM block was assessed at the follow-up visit of our pain clinic.

**Table 1:** Summary of symptoms meeting diagnostic criteria for MPS in three patients

Symptoms meeting diagnostic criteria for MPS	Case #1	Case #2	Case #3
Tender spots with referral of pain in the cervicoscapular region	Yes	Yes	Yes
Recognition of the cervicoscapular pain during palpation of tender spots	Yes	Yes	Yes
Cervicoscapular muscle stiffness	Yes	Yes	Yes
Limited range of motion of the shoulder joint	Yes	No	No
Pain worsens with stress	Yes	Yes	Yes

*MPS*, myofascial pain syndrome

**Table 2:** Characteristics and pharmacological treatments in three cases

	Case #1	Case #2	Case #3
Age (years)	67	49	61
Sex	Male	Female	Female
Height (cm)	171	152	162
Weight (kg)	80	99	65
Diagnosis	Cervicoscapular MPS Failed back surgery syndrome (lumbar)	Cervicoscapular MPS Lumbar hernia	Cervicoscapular MPS Failed back surgery syndrome (lumbar)
Pharmacological treatments (per day)	Diclofenac sodium 50 mg Tizanidine hydrochloride 3 mg Kakkon-to (herbal medicine) 7.5 g Hachimi-jio-gan (herbal medicine) 7.5 g	Acetaminophen 1800 mg Kakkon-to (herbal medicine) 5.0 g	Acetaminophen 1500 mg Tramadol 50 mg Codeine phosphate 12 mg Goshuyu-to (herbal medicine) 7.5 g

*MPS*, myofascial pain syndrome

### Case #1

**Fig. 1:**
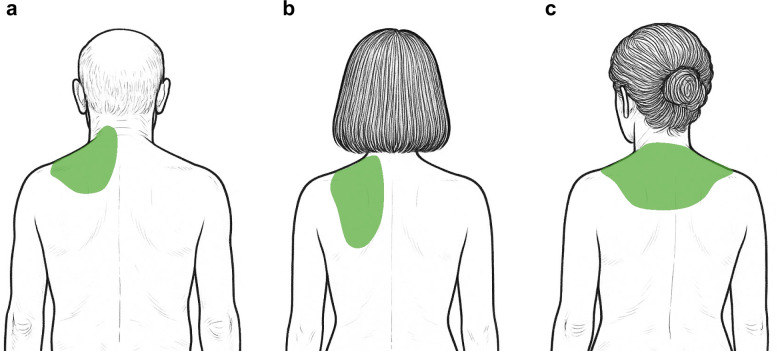
The location of pain in three myofascial pain syndrome patients presented with cervicoscapular pain. **a** Case #1, **b** Case #2, and **c** Case #3. The colored green area represents the location of the pain in each case

**Fig. 2:**
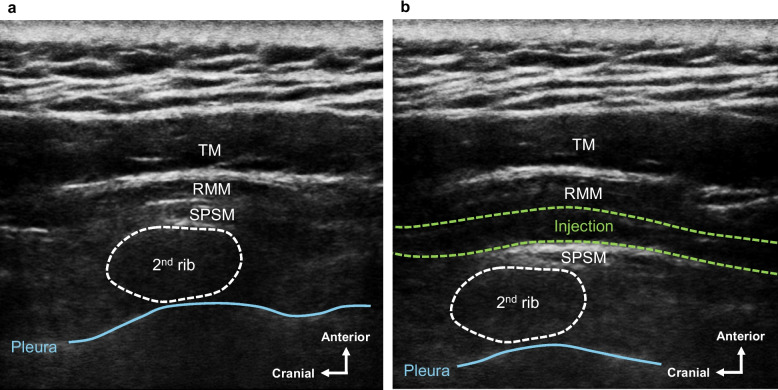
Ultrasound-guided serratus posterior superior muscle (SPSM) block **a** Ultrasound image of relevant anatomical structures in the simulated SPSM block in the cadaver. The dotted white lines show the boundary of the second rib. The blue line shows the pleura. **b** Ultrasound image following the SPSM block with administration of injection (30 mL of 0.25% ropivacaine) in case #1. The dotted white lines show the boundary of the second rib. The blue line shows the pleura. The dotted green lines show the boundaries of the administered injection. RMM, rhomboid major muscle; SPSM, serratus posterior superior muscle; TM, trapezius muscle.

A 67-year-old man, 171 cm tall, weighing 80 kg, has visited our pain clinic for more than 15 years for the diagnosis of cervicoscapular MPS and lumbar failed back surgery syndrome (FBSS). He complained of dull pain with a score of 10 on the 11-point NRS in the left cervicoscapular region (Fig. 1a), and his cervicoscapular pain was not sufficiently relieved by pharmacological treatments. Although multiple trigger point injections using 10 mL of a mixture of 1% lidocaine and vitamin B were dividedly administered to his left cervicoscapular region when the pain got worse, the pain relief continued only for 1–2 days after the procedures. On physical examination, active shoulder flexion was limited to 70°, extension to 10°, abduction to 30°, adduction to 0°, external rotation to 30°, and internal rotation to 50°, with pain at the end range. Passive shoulder range of motion was not evaluated at the request of the patient due to fear of cervicoscapular pain worsening. Ultrasound-guided SPSM block was performed under sterile conditions with the patient in the sitting position. After placing the linear probe (6–13 MHz) in the sagittal direction at the cervicoscapular spine level, we identified the second rib, the SPSM, rhomboid major muscle (RMM), and the trapezius muscle (TM) (Fig. 2a). After local anesthesia, an 80-mm 22-G non-insulated echogenic needle was inserted in the cranial direction using the in-plane technique, so that the needle contacted the surface of the SPSM at the second rib level. After the needle tip was confirmed by ultrasound as being in the appropriate space, 30 mL of 0.25% ropivacaine was applied between the SPSM and RMM (Fig. 2b). Sixty minutes after the SPSM block administration, the pain in his left cervicoscapular region decreased to an NRS score of 0, and the pain relief by the SPSM block expanded the range of motion of his shoulder, including active shoulder flexion to 150°, extension to 30°, abduction to 90°, adduction to 0°, external rotation to 50°, and internal rotation to 80°. No sensory loss was observed using the cold test. At the follow-up visit of our pain-clinic, which was 4 weeks after the SPSM block, the pain relief was confirmed to continue for 3 weeks after the SPSM block with 30 mL of 0.25% ropivacaine. The pain in his left cervicoscapular region at that time was evaluated as an NRS score of 10. After receiving the patient’s consent for performing the SPSM block with physiological saline, we performed the SPSM block with 30 mL of physiological saline on the left side. Sixty minutes after the SPSM block administration, the pain in his left cervicoscapular region decreased to an NRS score of 3. Naturally, no sensory loss was observed using the cold test. At the follow-up visit of our pain clinic, the pain relief was confirmed to continue for 3 weeks after the SPSM block with 30 mL of physiological saline.

### Case #2

A 49-year-old woman, 152 cm tall, weighing 99 kg, has visited our pain clinic for more than 15 years for the diagnosis of cervicoscapular MPS and lumbar hernia. She complained of dull pain with an NRS score of 2 in the left cervicoscapular region. When her cervicoscapular pain got worse with an NRS score of 6 (Fig. 1b), we provided her with 3 optional treatments including oral loxoprofen, multiple trigger point injections, and the SPSM block. After informing her of the benefits and the risks of each treatment, she finally chose the SPSM block. We performed the SPSM block with administration of 30 mL of 0.25% ropivacaine on her left side. Sixty minutes after the SPSM block administration, the pain in her left cervicoscapular region decreased to an NRS score of 1. No sensory loss was observed using the cold test. At the follow-up visit of our pain clinic, which was 4 weeks after the SPSM block, the pain relief was confirmed to continue for 2 weeks after the SPSM block administration with 30 mL of 0.25% ropivacaine.

### Case #3

A 61-year-old woman, 162 cm tall, weighing 65 kg, has visited our pain clinic for more than 15 years for the diagnosis of cervicoscapular MPS and lumbar FBSS. She complained of dull pain with an NRS score of 10 in her left and right cervicoscapular regions (Fig. 1c). Because both pharmacological treatments and multiple trigger point injections using 10 mL of a mixture of 1% lidocaine and vitamin B have not sufficiently relieved her cervicoscapular pain, we proposed to her that the SPSM block could be administered to her cervicoscapular regions instead of multiple trigger point injections. After receiving the patient’s consent, we performed the SPSM block with administration of 30 mL of physiological saline bilaterally. Sixty minutes after the SPSM block administration, the pain in her left and right cervicoscapular regions decreased to an NRS score of 3. Naturally, no sensory loss was observed using the cold test. At the follow-up visit of our pain clinic, which was 4 weeks after the SPSM block, the pain relief was confirmed to continue for 3 weeks after the SPSM block administration with 30 mL of physiological saline.

There were no complications concerned with the SPSM block in three patients. Injections of the SPSM block, changes in the NRS score, sensory loss, the pain relief period, and complications are summarized in Table 3.

**Table 3:** Injections of the SPSM block, changes in the NRS score, sensory loss, the pain relief period, and complications in three cases

	Case #1		Case #2	Case #3
Injection	30 mL of 0.25% ropivacaine	30 mL of physiological saline	30 mL of 0.25% ropivacaine	30 mL of physiological saline
NRS prior to the SPSM block	10	10	6	10
NRS 60 min after the SPSM block	0	3	1	3
Sensory loss in the dermatome between T1 and T8	None	None	None	None
The pain relief period (weeks)	3	3	2	3
Complication	None	None	None	None

*SPSM*, serratus posterior superior muscle;* NRS*, Numerical Rating Scale

## Discussion

The novelty of this case reports is that the SPSM block, which would act as an interfascial block rather than a segmental nerve block, using 30 mL of 0.25% ropivacaine or physiological saline, decreased an NRS score and provided 2–3 weeks of pain relief in MPS patients presented with cervicoscapular pain. Clinical guidelines for the MPS recommend multiple trigger point injections [1]. In multiple trigger point injections for the MPS, 1% lidocaine would be typically injected into the painful trigger points [7]. A small volume is considered the most effective, and less than 1 mL of 1% lidocaine should be divided and injected into each trigger point with a total volume of 10 mL of 1% lidocaine [7]. Therefore, we consider that the SPSM block, which would act as an interfascial block, completely differs from multiple trigger point injections for the MPS. Although the pathophysiology of MPS is still unknown, the histological fact was reported that the interfascial space contains nociceptive nerve branches that innervate the fascia [8–10]. Furthermore, we have previously reported that the SPSM block provides pain relief by acting as an interfascial block, and the SPSM block with 30 mL of injectate volume tends to spread more widely between the SPSM and RMM and penetrate into the RMM in the cadaveric study [5]. Therefore, we assumed that the SPSM block with 30 mL of local anesthetics could more effectively provide pain relief compared to multiple trigger point injections in MPS patients with cervicoscapular pain. In fact, the SPSM block with 30 mL of 0.25% ropivacaine drastically decreased an NRS score and provided 2–3 weeks of pain relief in cases #1 and #2. No sensory loss observed using the cold test in #1 and #2 suggested that the SPSM block would provide pain relief by acting as an interfascial block, not a segmental nerve block. We have also reported that the SPSM block with 30 mL of injectate volume tends to penetrate into the RMM, but not into the TM, rhomboid minor, SPSM, erector spinae, and intercostal muscles [5]. Seol et al. [11] previously reported that trigger point injection into the RMM, which is deeply related to musculoskeletal pain in the cervicoscapular and upper back region, provided pain relief in patients with cervicoscapular MPS. Therefore, the SPSM block with 30 mL of 0.25% ropivacaine might potentially provide rapid pain relief not only via an interfascial block, but also intramuscular block of the RMM in cases #1 and #2. On the contrary, the SPSM block with 30 mL of physiological saline also mildly decreased an NRS score and provided 3 weeks of pain relief in cases #1 and #3. This finding is consistent with the previous report that the interfascial blocks with injection of physiological saline between the affected muscles have reduced the intensity of pain in patients with MPS [2]. Furthermore, because the block procedures with the high dose of local anesthetics would contain the potential risk of local anesthetic toxic syndrome, the SPSM block with physiological saline would be preferable to that with local anesthetics in MPS patients with bilateral cervicoscapular pain. One of the possible pathophysiology of MPS was previously reported that tightened myofascial tissue with impaired sliding fascial mobility causing chronic musculoskeletal pain [12]. Therefore, we considered that the SPSM block with 30 mL of physiological saline effectively decreased cervicoscapular pain in MPS patients by causing wide myofascial release, which restores the sliding function and flexibility of the myofascial tissue. There are some limitations in the present case reports. First, we administered the SPSM block with only 30 mL of 0.25% ropivacaine in patients with MPS. We should investigate whether the lower concentration of ropivacaine in 30 mL volume would provide pain relief in MPS patients with cervicoscapular pain. Second, in the present cases, the SPSM block decreased the NRS score but did not lead to a reduction in the dose of the pharmacological treatments.

We presented the case reports in which the SPSM block successfully decreased the NRS scores in MPS patients. These findings suggest that the SPSM block may serve as a useful therapeutic option in MPS patients presenting with cervicoscapular pain.

## Data Availability

The data that support the findings of this case reports are available from the corresponding author [A.S.] upon reasonable request.

## Abbreviations

MPS: Myofascial pain syndrome
SPSM: Serratus posterior superior muscle
NRS: Numerical Rating Scale
FBSS: Failed back surgery syndrome
RMM: Rhomboid major muscle
TM: Trapezius muscle
